# Increases in Anxiety and Depression During COVID-19: A Large Longitudinal Study From China

**DOI:** 10.3389/fpsyg.2021.706601

**Published:** 2021-07-06

**Authors:** Shizhen Wu, Keshun Zhang, Elizabeth J. Parks-Stamm, Zhonghui Hu, Yaqi Ji, Xinxin Cui

**Affiliations:** ^1^Student Counselling and Mental Health Center, Qingdao University, Qingdao, China; ^2^Department of Psychology, Normal College, Qingdao University, Qingdao, China; ^3^Department of Psychology, University of Southern Maine, Portland, ME, United States; ^4^Department of Psychology, School of Social Science, The University of Manchester, Manchester, United Kingdom

**Keywords:** COVID-19, anxiety, depression, university students, longitudinal study

## Abstract

Although accumulating evidence suggests the COVID-19 pandemic is associated with costs in mental health, the development of students' mental health, including the change from their previous levels of depression and anxiety and the factors associated with this change, has not been well-studied. The present study investigates changes in students' anxiety and depression from before the pandemic to during the lockdown and identifies factors that are associated with these changes. 14,769 university students participated in a longitudinal study with two time points with a 6-month interval. Students completed the Anxiety and Depression subscales of the Symptom Checklist 90 (SCL-90) before the COVID-19 outbreak (October 2020, Time 1), and the Self-rating Anxiety Scale (SAS) and Self-rating Depression Scale (SDS) during the pandemic (April 2020, Time 2). The prevalence of anxiety and depression symptoms were 1.44 and 1.46% at Time 1, and 4.06 and 22.09% at Time 2, respectively, showing a 181.94% increase in anxiety and a 1413.01% increase in depression. Furthermore, the increases in anxiety and depression from pre-pandemic levels were associated with students' gender and the severity of the pandemic in the province where they resided. This study contributes to the gap in knowledge regarding changes in students' mental health in response to the pandemic and the role of local factors in these changes. Implications for gender and the Typhoon Eye effect are discussed.

## Introduction

The COVID-19 global pandemic caused a large number of infections and deaths (Cucinotta and Vanelli, 2020). To contain the virus, China initiated a series of emergency management steps at the beginning of March 2020, including shutting down schools and initiating online learning for close to 30 million university students across the country (i.e., Suspending Classes without Stopping Learning, http://www.moe.gov.cn/). Researchers around the world have called for researchers to examine the impact of the pandemic and school closures on students' anxiety, depression, and other outcomes (Holmes et al., 2020). Research suggests that the combined psychological pressure caused by the pandemic and the quarantine heightened anxiety and depression, particularly among university students (e.g., Brooks et al., 2020; Peng et al., 2020; Zhang et al., 2021).

Research around the world has reported high prevalence rates of anxiety and depression symptoms during the COVID-19 outbreak (Ahmed et al., 2020; Peng et al., 2020; Xiong et al., 2020). In a survey conducted from January 31 to February 3, 2020, nearly 30% of university students reported anxiety symptoms and more than 20% reported depression symptoms (Chang et al., 2020). A systematic review and meta-analysis conducted by Salari et al. (2020) estimated that the worldwide prevalence of anxiety and depression in the general population after the outbreak of COVID-19 was 31.9 and 33.7%, respectively. The costs of this heightened anxiety and depression for students are manifold, including impacts on students' thinking, motivation, interpersonal communication, and physical health; and could lead to sleep disturbances, loss of appetite, and even self-harm (e.g., Ystgaard et al., 1999; Gotlib and Hammen, 2008; Felger et al., 2015; Oxford, 2015).

Research conducted early in the pandemic suggested that individuals' level of anxiety and depression may be related to the province where they lived, and especially to the number of confirmed cases in the city (e.g., Ho et al., 2020; Xiong et al., 2020; Zhao et al., 2020). Past theorizing on the psychological effects of proximity to disaster offers predictions about how individuals' residence should impact their psychological well-being. Based on the impact of the Wenchuan earthquake on individuals' concerns about safety and health, Li et al. (2009, 2010) coined the term “Psychological Typhoon Eye” effect. This term refers to a pattern—like being in the eye of a storm—in which individuals who are closest to the center of the devastated area paradoxically show the least damaging psychological effects (Li et al., 2009, 2010). This pattern of results has been observed in negative correlations between schoolchildren's proximity to Ground Zero following the 9–11 attacks and their psychological well-being (Hoven et al., 2005) and between the level of exposure to SARS and anxiety (Xie et al., 2011). Zhang et al. (2020) found support for the Typhoon Eye effect in individuals' psychological responses to the COVID-19 pandemic, showing a negative correlation between the exposure level in 31 provinces in China and reported cases of mental health problems. However, others have found contradictory results. Zhao et al. (2020) reported that people who lived in high epidemic areas (provinces with more than 800 confirmed cases before Feb. 6, 2020) showed *greater* increases in anxiety from a baseline measure than those in low epidemic areas (i.e., other provinces in mainland China). Thus, in the present study we enter this debate by examining the relationship between the severity of the epidemic in the provinces where university students live (while completing classes remotely) and their anxiety and depression, controlling for their pre-pandemic levels.

A second factor that may influence anxiety and depression during the COVID-19 pandemic is gender (Ben-Ezra et al., 2020; Duan et al., 2020; Tang et al., 2020). Previous studies have found significant gender differences in anxiety and depression levels during the pandemic (Elbay et al., 2020; Mazza et al., 2020). A systematic review and meta-analysis found women suffered more severe anxiety and depression symptoms than men during the COVID-19 epidemic (Salari et al., 2020). Zhu et al. (2020), on the other hand, reported that males were more likely to experience depression in response to the pandemic. Other studies have found no significant differences in anxiety and depression levels between men and women (Chi et al., 2020; Van Rheenen et al., 2020). Therefore, this study will test how gender influences the development of anxiety and depression among college students in response to the COVID-19 epidemic, from before the start of the pandemic to during the lockdown. We further explore demographic factors previously known to be risk factors for anxiety and depression among college students, including economic status and medical conditions (e.g., Eisenberg et al., 2007; Xiong et al., 2020; Xu et al., 2020; Zhao et al., 2020).

In sum, our study has two aims. Using a longitudinal design, we first estimate the changes in anxiety and depression experienced by students from before the pandemic to during lockdown. Second, we examine whether the level of epidemic severity in students' geographic location, along with demographic variables like gender, economic status, and medical status, impact the change observed in anxiety and depression during the pandemic, as a test of the Typhoon Eye effect.

## Method

### Ethical Statement

This study complied with the ethical standards of the Declaration of Helsinki. All procedures were approved by our university's Research Ethics Committee. All participants willingly gave their informed consent to participate after being informed about the purpose of the study. All analyses were based on anonymous data.

### Participants and Design

Longitudinal data were collected via a Chinese online research panel, Wenjuanxing (https://www.wjx.cn/). Twenty-four thousand six hundred ninety-six university students participated in Time 1 (T1) assessment, and 14,769 of these participants took part in the Time 2 (T2) assessment (8,060 female, 6,709 male), with a 40.14% attrition rate. Anxiety and depression did not differ between those who completed the second assessment and those who did not. The age of participants ranged from 17 to 34 years (*M* = 20.76, *SD* = 1.97).

T1 took place in October 2019 (before the COVID-19 outbreak) and T2 took place in April 2020 (when students were completing remote learning from home due to the pandemic). Both T1 and T2 focused on students' anxiety and depression. Different instruments for anxiety and depression were used at the two time points.

### Measures

#### Anxiety and Depression of Symptom Checklist 90 (SCL-90)

We applied the subscale of the SCL-90 (Derogatis et al., 1973; Wang, 1984) to assess anxiety and depression in T1, which contains 10 items and 13 items, respectively. The items were rated along a 5-point response scale with 1-5 representing the severity as follows: “1 = no”, “2 = light”, “3 = moderate,” “4 = quite heavy,” and “5 = severe.” A standardized scoring algorithm is used to define anxiety symptoms, with a total score range of 10–50. Individuals were categorized as experiencing anxiety symptoms if the anxiety subscale score was >20. A standardized scoring algorithm was similarly used to define depression symptoms, with a total score range of 13–65. Individuals were categorized as experiencing depression symptoms if the depression subscale score was >26. The anxiety and depression subscales were internally consistent (Cronbach's *α*_t1_ = 0.85 and 0.89, respectively).

#### Zung Self-Rating Anxiety Scale

Anxiety at T2 was measured by the Chinese version of the SAS (Zung, 1971; Wu, 1999). The scale covers both psychological (e.g., “I feel afraid for no reason at all”) and somatic (e.g., “My arms and legs shake and tremble”) aspects of participants' anxiety symptoms. The items were rated along a 4-point response scale ranging from 1 (a little of the time) to 4 (most of the time). A standardized scoring algorithm is used to define anxiety symptoms, with a total score range of 25–100. Individuals were categorized as experiencing anxiety symptoms if the SAS score was greater than or equal to 50. The scale was internally consistent (Cronbach's α = 0.77).

#### Zung Self-Rating Depression Scale

Depression at T2 was measured by the Chinese version of SDS (Zung, 1965; Shu, 1999). It contains 20 items (e.g., “I have trouble sleeping at night,” “I get tired for no reason”) based on the diagnostic criteria of depression. Participants responded using a 4-point Likert scale ranging from 1 (a little of the time) to 4 (most of the time). A standardized scoring algorithm was used to define depression symptoms, with a total score range of 25–100. Individuals were categorized as experiencing depression symptoms if the SDS score was greater than or equal to 50. The scale was internally consistent (Cronbach's α = 0.86).

#### Epidemic Area

The epidemic area was defined as the cumulative number of confirmed cases in the province through the end of April 2020. Areas with 1–99 confirmed cases were labeled low (Level 1), areas with 100–999 confirmed cases were labeled middle (Level 2), and areas with more than 1,000 confirmed cases were labeled high epidemic areas (Level 3).

#### Demographics

Demographics included general demographic variables and economic status. The general demographic variables included gender and age. The economic status-related variables included per capita disposable income, per capita consumption expenditure, and the general public healthcare budget of each province. The data used was retrieved from the 2019 China Statistical Yearbook, which was published by China Statistics Press (http://www.stats.gov.cn/tjsj/ndsj/2019/indexch.htm).

### Data Analysis

The statistical analyses were performed using SPSS Version 25.0 and Python. The stats. ks_2samp method was used to test the distribution of variables (Hodges, 1958; The Scipy Community, 2020). The 95% bias-corrected confidence interval (95% CI) was set, and the statistical significance level was set at *p* < 0.05. Latent profile analyses (LPA) were conducted in R (version 4.10.1) using the package tidyLPA, dplyr, and tidyverse to classify anxiety and depression in T1 and T2. Akaike information criterion (AIC), Bayesian information criterion (BIC), and entropy (range from 0 to 1) were applied as criteria (Schwarz, 1978; Akaike, 1987). Lower AIC and BIC values indicate better model fit, while higher entropy values indicate greater certainty.

## Results

### Common Method Biases

All the participant variables involved in this study were collected by online questionnaire, and a Harman single-factor test was used to diagnose the common method bias (Podsakoff et al., 2003). The results of principal component factor analysis without rotation showed that there were 22 factors whose eigenvalues were >1. The variance explained by the first factor was 20.15%, which falls below the threshold of 40%. This result indicates that there is no serious common method bias in this study.

### Descriptive Statistics and Correlations

The descriptive results for anxiety and depression are shown in Table 1. The prevalence rate of anxiety and depression symptoms were 1.44 and 1.46% at T1, and 4.06 and 22.09% at T2, respectively, which represented a 181.94% increase in anxiety and a 1413.01% increase in depression.

**Table 1:** The proportion of different levels of anxiety and depression.

	**T1**				**T2**			
	***M***	***SD***	**n**	**%**	***M***	***SD***	**n**	**%**
No anxiety	13.60	3.91	14,556	98.56	35.10	6.21	14,170	95.94
Anxiety	33.69	4.10	212	1.44	57.06	5.95	599	4.06
No depression	17.59	5.42	14,553	98.54	37.84	6.47	11,507	77.91
Depression	44.35	5.53	216	1.46	58.91	5.33	3,262	22.09

**Figure 1:**
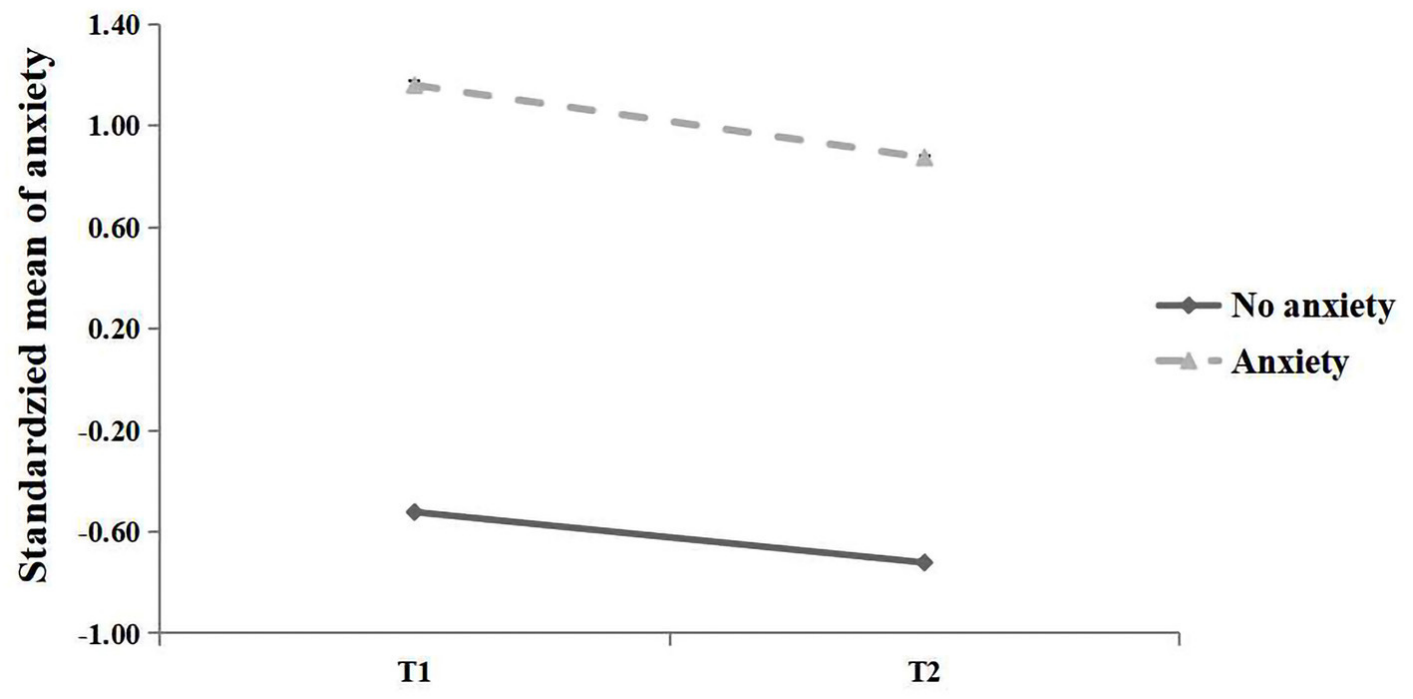
Standardized mean of anxiety for two latent profiles at T1 and T2.

**Figure 2:**
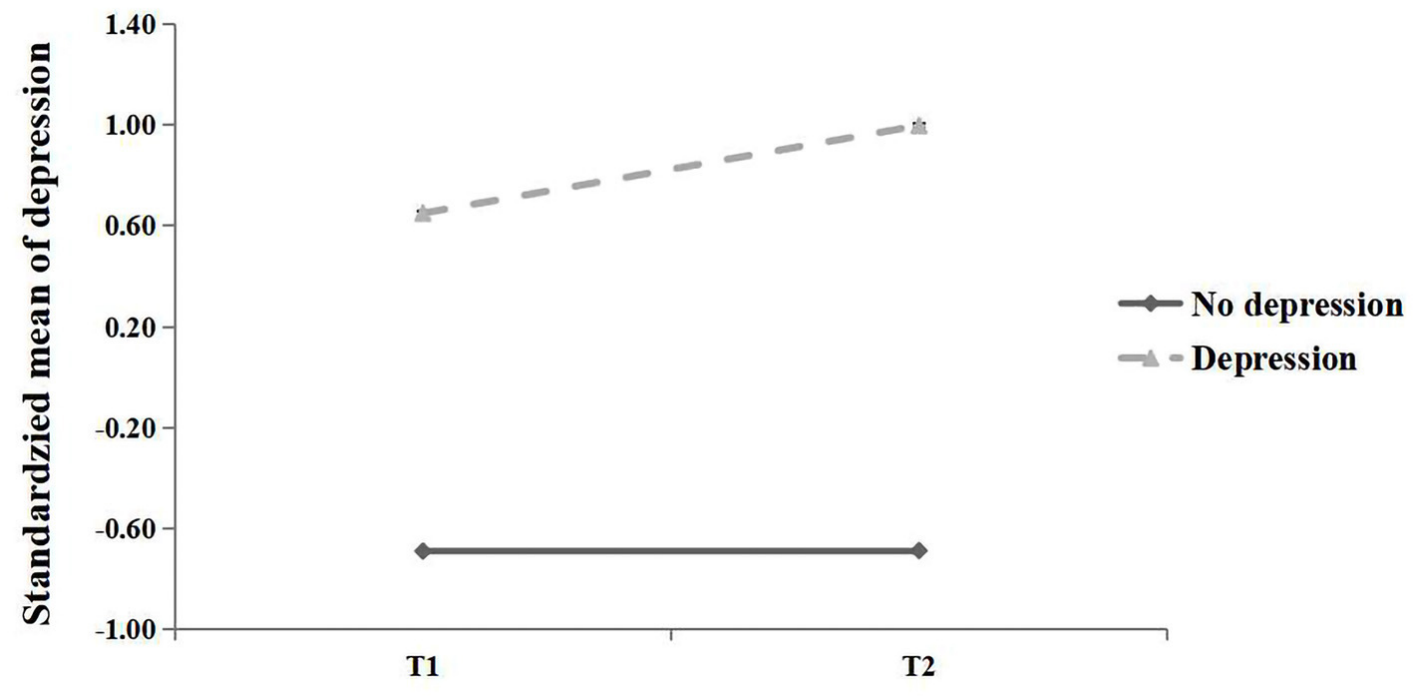
Standardized mean of depression for two latent profiles at T1 and T2.

A latent profile analysis (LPA) was conducted to explore anxiety and depression as categorical variables. The LPA results indicated that two profiles of anxiety and depression can be best distinguished with best class model fittings (Table 2). Figure 1 presents the standardized mean of anxiety of two profiles at T1 and T2. Profile 1 was characterized by significantly lower mean cores than Profile 2 at both time points, and were thus labeled *no anxiety* and *anxiety*, respectively. Figure 2 presents the standardized mean of depression of two profiles at T1 and T2. Profile 1 was characterized by significantly lower mean cores than Profile 2 at both time points, and were thus labeled *no depression* and *depression*, respectively.

**Table 2:** Summary of AIC, BIC, and Entropy values for latent profile models.

**Variable**	**Number of profiles**	**AIC**	**BIC**	**Entropy**
T1 anxiety	1	41915.61	41930.81	1.00
	2	36883.89	36914.29	0.94
	3	36887.89	36933.50	0.42
T2 anxiety	1	41915.61	41930.81	1.00
	2	40537.86	40568.26	0.92
	3	40541.82	40587.42	0.36
T1 depression	1	41915.61	41930.81	1.00
	2	36746.11	36776.51	0.92
	3	36750.13	36795.74	0.42
T2 depression	1	41915.61	41930.81	1.00
	2	39663.58	39693.98	0.79
	3	39226.52	39272.12	0.71

*AIC, Akaike's information criterion; BIC, Bayesian information criterion; Entropy values range from 0 to 1*.

Pearson correlations between variables are displayed in Table 3. Anxiety and depression were correlated with each other at T1 (*r* = 0.83, *p* < 0.01) and at T2 (*r* = 0.73, *p* < 0.01). Per capita disposable income and per capita consumption expenditure were negatively correlated with anxiety and depression at T1 and T2 (*r*s = −0.08 ~ −0.04, *p*s < 0.01). General public healthcare budget was negatively correlated with T2 anxiety (*r* = −0.02, *p* < 0.01). Men reported lower levels of depression than women at T1 (*t* = 3.23, *p* < 0.01) and higher levels of anxiety than women at T2 (*t* = −4.97, *p* < 0.001).

**Table 3:** Correlation between variables of this study (*N* = 14769).

	***M***	***SD***	**1**	**2**	**3**	**4**	**5**	**6**	**7**	**8**
T1 anxiety	13.89	4.59	–							
T1 depression	17.98	6.30	0.83^**^	–						
T2 anxiety	35.99	7.56	0.21^**^	0.24^**^	–					
T2 depression	42.50	10.73	0.19^**^	0.24^**^	0.74^**^	–				
Gender	–	–	−0.02	−0.03^**^	0.04^**^	0.01	–			
Per capita disposable income	28512.20	4369.83	−0.05^**^	−0.05^**^	−0.04^**^	−0.04^**^	0.07^**^	–		
Per capita consumption expenditure	37488.64	10924.20	−0.06^**^	−0.07^**^	−0.06^**^	−0.08^**^	0.06^**^	0.36^**^	–	
General public budget of health care	490.63	158.69	−0.01	−0.10	−0.02^*^	−0.02	0.03^**^	0.47^**^	−0.01	–

* *p < 0.05*,

** *p < 0.01*.

*T1 anxiety range from 10 to 50. T1 depression range from 13 to 65. T2 anxiety range from 25 to 90. T2 depression range from 25 to 100. Per capita disposable income ranges from 17286.1 to 64182.6. Per capita consumption expenditure ranges from 11520.2 to 43351.3. General public budget of health care ranges from 105.55 to 1407.51. The unit of per capita disposable income and per capita consumption expenditure is yuan. The unit of general public budget of health care is thousand yuan*.

### Anxiety

All continuous variables were standardized in the repeated analysis. The results indicated that students reported higher levels of anxiety at T2 than T1 (*β* = 0.080, *F* = 5.95, *p* < 0.05, 95% CI [0.016, 0.144]), with significantly different distributions of anxiety (Kolmogorov-Smirnov test; *Z* = 0.198, *p* < 0.001).

**Figure 3:**
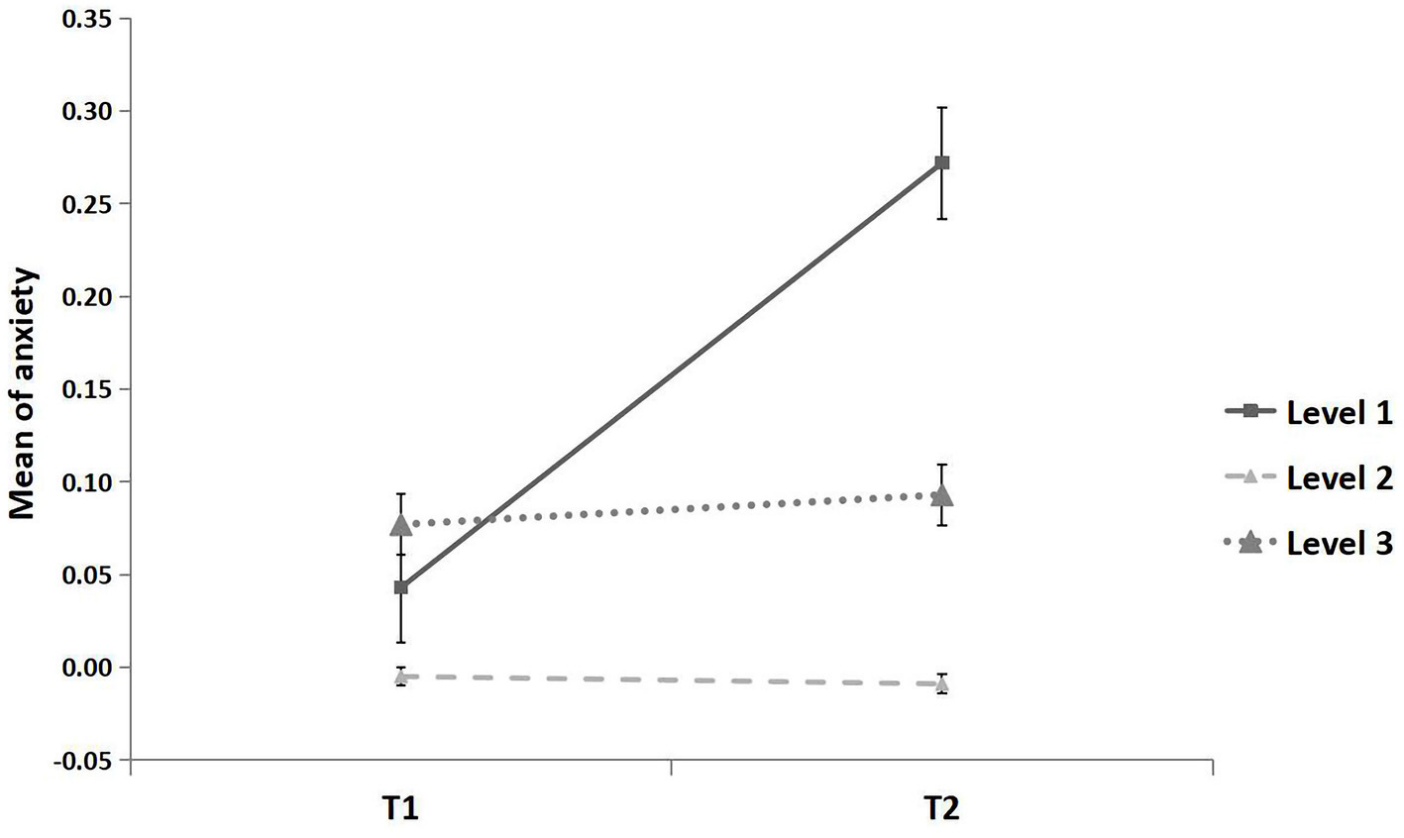
Mean of anxiety for three epidemic areas. All continuous variables were standardized. The epidemic area was defined as the cumulative number of confirmed cases in each province until the end of April 2020, i.e., low epidemic area (level 1): 1–99 cases, middle epidemic area (level 2): 100–999 cases, high epidemic area (level 3): ≥1,000 cases. Error bar is the standard error.

**Figure 4:**
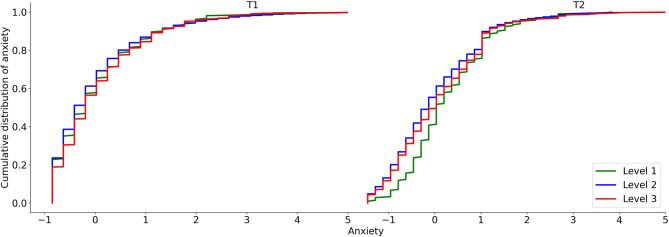
The cumulative distribution of anxiety for the three epidemic areas.

The effect of time on anxiety was significantly moderated by epidemic area level (*F* = 3.67, *p* < 0.05; Figure 3). In line with the Typhoon Eye effect, the mean increase in anxiety from T1 to T2 was significantly lower in the areas with the highest severity level (*MD*_(*T*2−*T*1)_ = 0.004, *p* = 0.682, 95% CI [−0.017, 0.025]) than the increase among those in the lowest severity areas (*MD*_(*T*2−*T*1)_ = 0.229, *p* < 0.01, 95% CI [0.060, 0.398]), qualified by significantly different distribution at T2 (Kolmogorov-Smirnov test; *Z* = 0.15, *p* < 0.001; Figure 4), and not significantly different from areas with a moderate severity level (*MD*_(*T*2__−*T1)*_ = 0.016, *p* = 0.730, 95% CI [−0.074, 0.106]).

**Figure 5:**
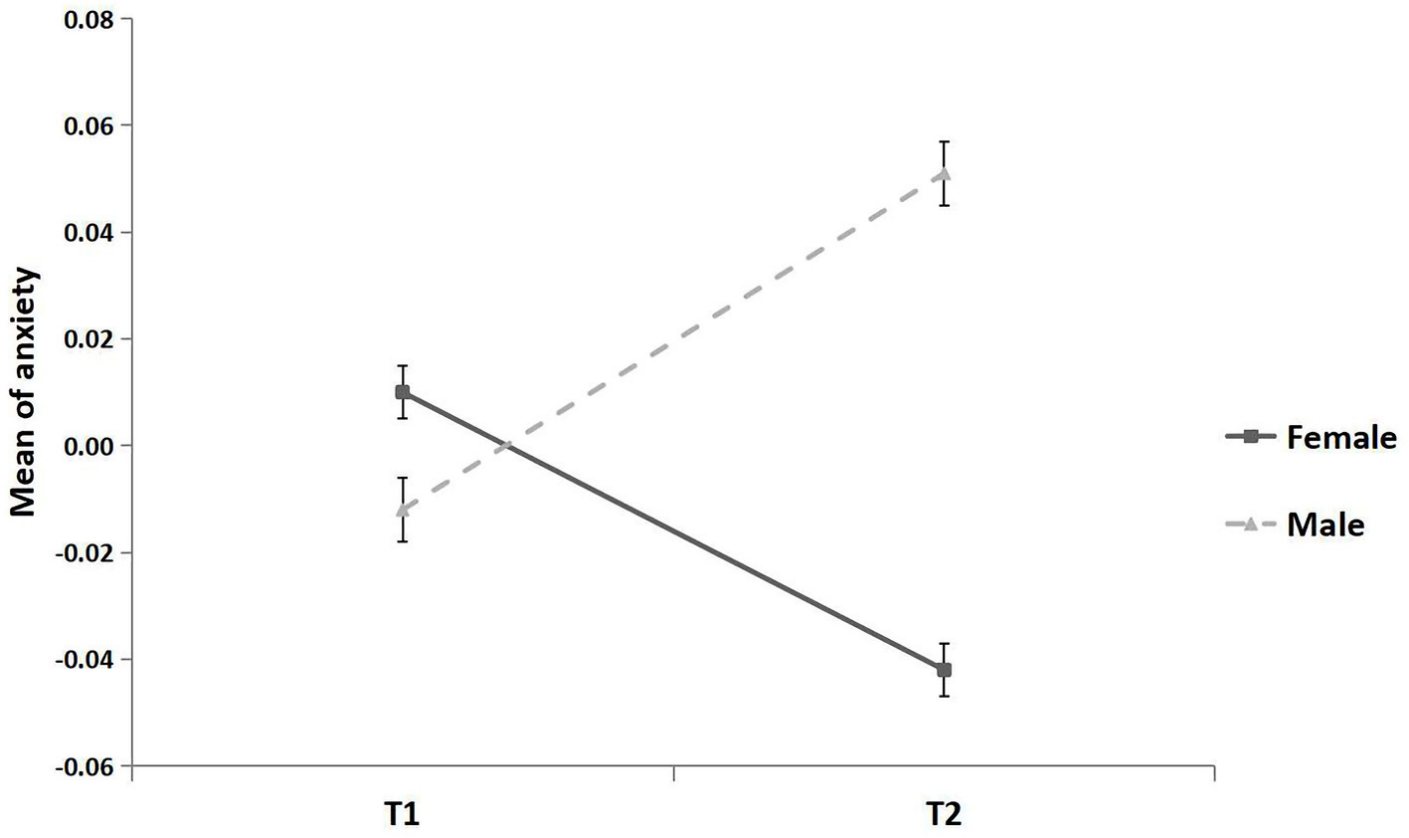
Mean of anxiety for females and males. All continuous variables were standardized. Error bar is the standard error.

The effect of gender on anxiety was significantly moderated by time (*F* = 30.35, *p* < 0.001; Figure 5). At T1, no gender differences in anxiety were found (*t* = 1.85, *p* = 0.06). At T2, males reported significantly higher anxiety than females (*β* = 0.082, *p* < 0.001, 95% CI [0.050, 0.114]).

We used logistic regression to assess the effects of T1 anxiety, T1 depression, epidemic level of the area, economic-related variables, and gender on T2 anxiety (Table 4). T1 anxiety (profile 1: no anxiety = 0; profile 2: anxiety = 1), T1 depression (profile 1: no depression = 0; profile 2: depression = 1), T2 anxiety (profile 1: no anxiety = 0; profile 2: anxiety = 1), the gender (male = 1, female = 0), and epidemic area (comparing Level 1 vs. Level 3 [Level 1_3]: Level 1 = 1, level 3 = 0; comparing Level 2 vs. Level 3 [Level 2_3]: Level 2 = 1, Level 3 = 0) were dummy coded. The regression model was significant, [*F*_(8, 14760)_ = 55.73, *p* < 0.001, *R*^2^ = 0.029]. The regression coefficients of T1 anxiety (*β* = 0.083, *t* = 7.94, *p* < 0.001), T1 depression (*β* = 0.096, *t* = 0.93, *p* < 0.001), gender (*β* = 0.044, *t* = 5.39, *p* < 0.001), per capita consumption (*β* = −0.022, *t* = −4.20, *p* < 0.001) and Level 1 vs. 3 (*β* = 0.081, *t* = 2.04, *p* < 0.05) were significant.

**Table 4:** The regression model of T2 anxiety.

	***B***	***SE***	***t***	***p***	**95% CI**
Constant	0.362	0.022	16.517	<0.001	[0.319, 0.405]
T1 depression	0.096	0.010	9.928	<0.001	[0.077, 0.115]
T1 anxiety	0.083	0.010	7.941	<0.001	[0.063, 0.104]
Level 1_3	0.081	0.040	2.039	<0.05	[0.003, 0.158]
Level 2_3	−0.008	0.022	−0.351	0.726	[−0.050, 0.035]
Gender	0.044	0.008	5.385	<0.001	[0.028, 0.060]
Per capita disposable income	0.002	0.005	0.358	0.720	[−0.009, 0.012]
Per capita consumption expenditure	−0.022	0.005	−4.203	<0.001	[−0.033, −0.012]
General public healthcare budget	−0.010	0.005	−2.041	0.041	[−0.019, 0.000]

*All continuous variables were standardized; T1 anxiety: no anxiety = 0, anxiety = 1, T1 depression: no depression = 0, depression = 1, T2 anxiety: no anxiety = 0, anxiety = 1; Level 1_3: Level 1 = 1, level 3 = 0; Level 2_3: Level 2 = 1, Level 3 = 0*.

### Depression

All continuous variables were standardized in the repeated analysis. The results showed that students were more depressed at T2 than T1 (β = 0.075, *F* = 5.35, *p* < 0.05, 95% CI [0.11, 0.138]), with significantly different distributions of depression (Kolmogorov-Smirnov test; *Z* = 0.265, *p* < 0.001).

**Figure 6:**
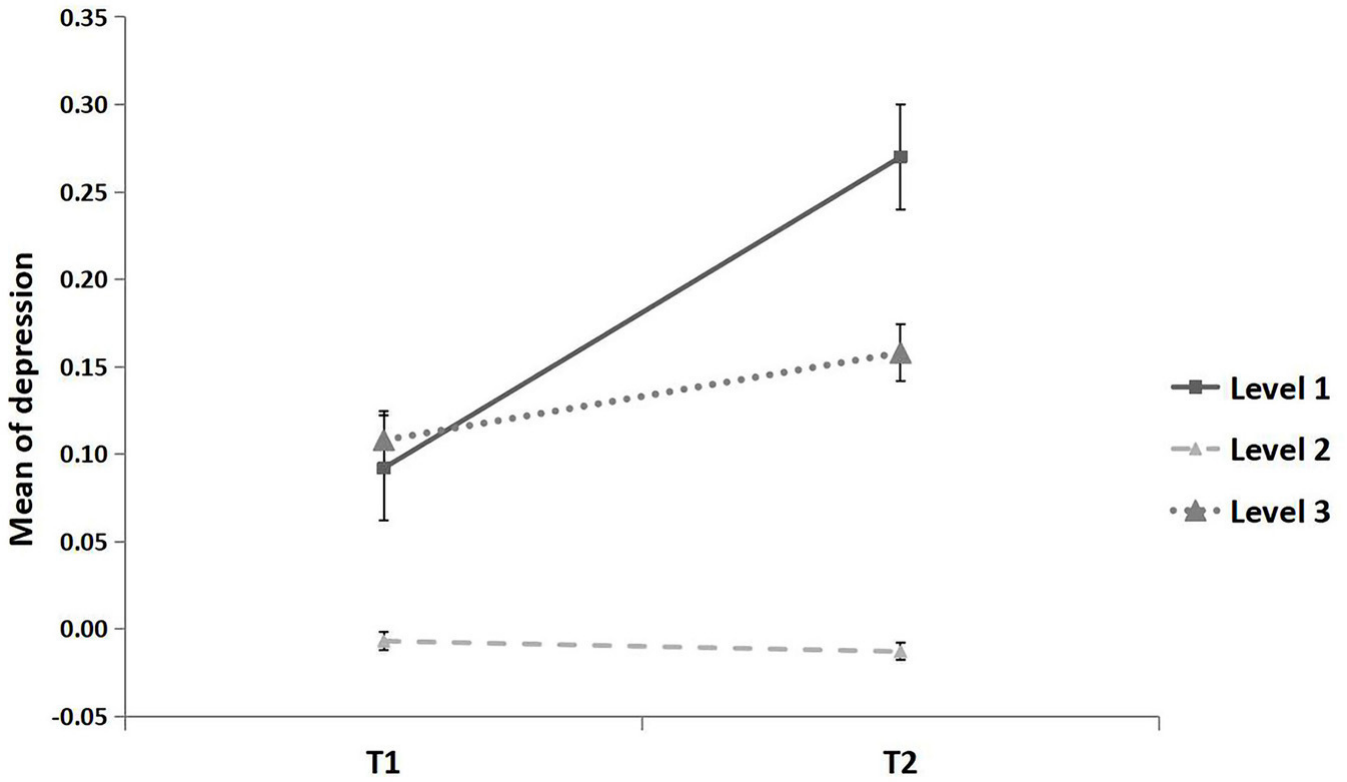
Mean of depression for three epidemic areas. All continuous variables were standardized. The epidemic area was defined as the cumulative number of confirmed cases in each province until the end of April 2020, i.e., low epidemic area (level 1): 1–99 cases, middle epidemic area (level 2): 100–999 cases, high epidemic area (level 3): ≥1,000 cases. Error bar is the standard error.

**Figure 7:**
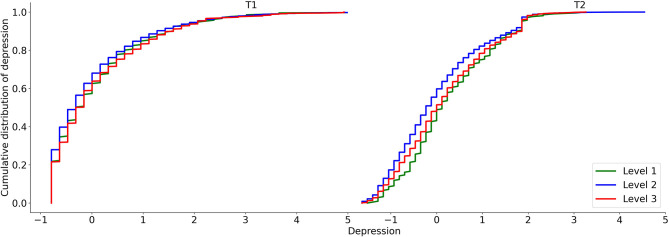
The cumulative distribution of depression for the three epidemic areas.

The effect of time on depression was significantly moderated by the area epidemic level (*F* = 2.98, *p* = 0.05; see Figure 6). Again, in line with the Typhoon Eye effect, the mean increase in depression from T1 to T2 was significantly lower in the areas with the highest severity level (*MD*_(*T*2__−*T1)*_ = 0.051, *p* = 0.264, 95% CI [−0.038, 0.139]) than the increase in the lowest severity level (*MD*_(*T*2__−*T1)*_ = 0.179, *p* < 0.05, 95% CI [0.012, 0.345]), although this was not significant in term of distribution (Kolmogorov-Smirnov test; *Z* = 0.08, *p* = 0.20; Figure 7), and was not significantly different from areas with a moderate severity level (*MD*_(*T*2__−*T1)*_ = −0.005, *p* = 0.603, 95% CI [−0.026, 0.015]).

**Figure 8:**
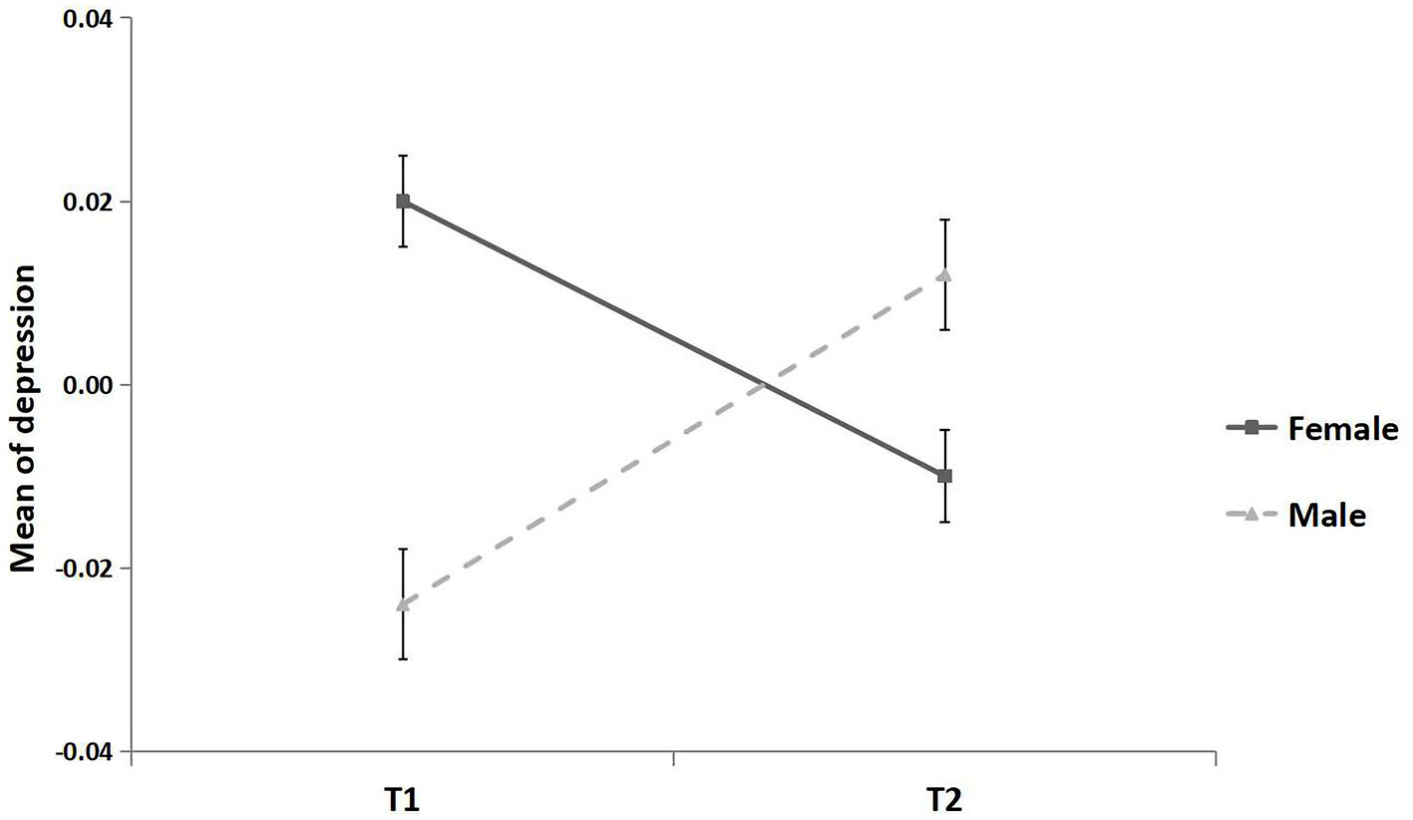
Mean of depression for females and males. All continuous variables were standardized. Error bar is the standard error.

The effect of gender on depression was significantly moderated by time (*F* = 10.52, *p* < 0.001; see Figure 8). At T1, females reported higher levels of depression than males (β = 0.054, *p* < 0.005, 95% CI [0.021, 0.086]). At T2, the mean levels of depression did not differ between male and female (*t* = −0.64, *p* = 0.52).

We used logistic regression to assess the effects of T1 anxiety, T1 depression, epidemic level of the area, economic-related variables, and gender on T2 depression (Table 5). T1 anxiety (profile 1: no anxiety = 0; profile 2: anxiety = 1), T1 depression (profile 1: no depression = 0; type 2: depression = 1), T2 depression (profile 1: no depression = 0; profile 2: depression = 1), the gender (male = 1, female = 0), and epidemic area (comparing Level 1 vs. Level 3 [Level 1_3]: Level 1 = 1, level 3 = 0; comparing Level 2 vs. Level 3 [Level 2_3]: Level 2 = 1, Level 3 = 0) were dummy coded. The regression was significant, [*F*_(8, 14760)_ = 66.48, *p* < 0.001, *R*^2^ = 0.035]. As shown in Table 4, the regression coefficients of T1 depression (*β* = 0.126, *t* = 13.15, *p* < 0.001), T1 anxiety (*β* = 0.068, *t* = 6.64, *p* < 0.001) and per capita consumption (*β* = −0.026, *t* = −5.02, *p* < 0.001) were significant.

**Table 5:** The regression model of T2 depression.

	***B***	***SE***	***t***	***p***	**95% CI**
Constant	0.337	0.022	15.618	<0.001	[0.295, 0.380]
T1 depression	0.126	0.010	13.148	<0.001	[0.107, 0.144]
T1 anxiety	0.068	0.010	6.636	<0.001	[0.048, 0.089]
Level 1_3	0.018	0.039	0.472	0.637	[−0.058, 0.095]
Level 2_3	−0.022	0.021	−1.029	0.303	[−0.064, 0.020]
Gender	0.011	0.008	1.377	0.169	[−0.005, 0.027]
Per capita disposable income	0.000	0.005	0.058	0.954	[−0.010, 0.011]
Per capita consumption expenditure	−0.026	0.005	−5.018	<0.001	[−0.036, −0.016]
General public healthcare budget	−0.003	0.005	−0.640	0.522	[−0.012, 0.006]

*All continuous variables were standardized; T1 anxiety: no anxiety = 0, anxiety = 1, T1 depression: no depression = 0, depression = 1, T2 depression: no depression = 0, depression = 1; Level 1_3: Level 1 = 1, level 3 = 0; Level 2_3: Level 2 = 1, Level 3 = 0*.

## Discussion

Through measures of anxiety and depression both before and after the pandemic outbreak, we found that the prevalence of university students with anxiety and depression symptoms above the standardized threshold increased 2.62 and 20.63%, respectively, to 4.06 and 22.09%. Furthermore, the increases in anxiety and depression were significantly moderated by both the severity level of the COVID-19 pandemic of the province where they lived and with their gender. In line with the Typhoon Eye effect, students living in areas with the least cases reported the greatest increase in anxiety and depression. In a second major finding, men's anxiety and depression increased more than women's during the lockdown.

The significant increase in the prevalence of both anxiety and depression symptoms were consistent with our expectations regarding the impact of the COVID-19 outbreak on university students' mental health. Other researchers have found that anxiety and depression have been heightened during the pandemic, particularly among quarantined individuals (Tang et al., 2020; Wang Y. et al., 2020). The shift to remote learning may have been especially challenging for university students, as living in a dorm has been found to be a protective factor for mental health problems (Eisenberg et al., 2007). In line with research showing the negative effects of reduced social support on alcohol abuse during remote learning (Lechner et al., 2020), removing students from this important source of social support may have exacerbated the effect of the pandemic on college students' mental health. The latent profile analysis additionally showed that it was the most vulnerable students—those who reported higher levels of anxiety and depression at time 1—who showed the greatest increases in these symptoms during remote learning.

The current study breaks new ground in examining how the severity of the pandemic in one's geographic region impacts students' anxiety and depression. Our results support the Psychological Typhoon Eye effect in that individuals' mental state in the areas with the highest epidemic level—the eye of the storm–was relatively calm (Li et al., 2010; Wang G. et al., 2020). This mechanism underlying this important finding should be examined by future research. Our study highlights a potential explanation for this seemingly paradoxical effect: individuals' perception of public and governmental support. For example, citizens living in high epidemic areas such as Wuhan received medical staff and facility support from all over China, whereas citizens in the low epidemic areas such as Tibet, Inner Mongolia, and Guangxi province may have perceived their medical support systems to be more vulnerable. This could dampen the level of anxiety and depression in high epidemic areas but exacerbate them in low epidemic areas (Xie et al., 2011; Zheng et al., 2015; Zhang et al., 2020). In our results, we see this in the negative relationship between the public healthcare budget in an area and citizens' levels of anxiety during the pandemic. Another mechanism that may explain the higher anxiety and depression outside the “eye” of the pandemic is the role of social media and news for university students. Consuming social media and news related to the pandemic has been found to worsen individuals' anxiety and depression (Gao et al., 2020; Li et al., 2020), whereas having direct experience with a hazard makes it appear less risky (Maderthaner et al., 1978). By studying the Typhoon Eye effect among a large sample of university students with pre-pandemic measures of anxiety and depression, the present study contributes important knowledge to a question that had previously shown contradictory results in the COVID-19 pandemic (Zhang et al., 2020; Zhao et al., 2020).

Gender has previously been identified as one of the predictive factors of mental health during the pandemic. In recent studies examining the general population, including individuals involved in retail, the service industry, and healthcare, women tended to be more likely to develop symptoms of anxiety and depression than men (e.g., Lei et al., 2020; Xiong et al., 2020). However, our results indicated that the increase in anxiety and depression during the lockdown was greater for male students than female students. We suggest two potential explanations for these findings. First, the switch to remote learning for university students during the lockdown means that students must live in a relatively closed environment with their parents while completing their online learning tasks, leading to parent-child conflict (Luo, 2020). Several lines of research indicate that male students experience more parent-child conflict than female students (Burt et al., 2006; Dotterer et al., 2008; Juang et al., 2012), and that parent-child conflict is associated with depression and anxiety (Marmorstein and Iacono, 2004; Lamis and Jahn, 2013). During the home quarantine, parent-child conflict and long-term exposure to adverse family emotional environments could be sources that caused the gender differences in mental health (Dunsmore and Halberstadt, 1997; Weymouth et al., 2016). A second potential explanation relates to gender differences in resiliency and coping. From an emotional coping perspective, a large number of previous studies have shown that men exhibit less expressive emotional behaviors than women (Barrett et al., 1998; Hess et al., 2000; Parkins, 2012; Chaplin and Aldao, 2013). Compared with men, women report more emotion-focused coping methods, including venting, emotional expression, and seeking social support (Billings and Moos, 1984; Ptacek et al., 1994), which may have enabled female students to adapt to the stressful environment more effectively (Cohen, 2004). Within the Chinese culture, males are also expected to exhibit greater expressive suppression than females (Cheng et al., 2009; Flynn et al., 2010; Zhao et al., 2014). Although expressive suppression can reduce the expression of negative emotions, it can have negative effects on cognition and emotion and is not an effective approach to emotion regulation (Gross and Levenson, 1997; Richards and Gross, 2000). Future research should explore how parent-child conflict and emotional suppression/expression differentially impacts male and female university students' coping with stress. The present findings imply that decisions to shut universities (to shift to remote learning e.g., in times of crisis) may be particularly harmful for male university students' mental health.

There are also important practical implications of the present work, which could be applied by universities and mental health counselors. The present study indicated that the pandemic has a negative impact on the anxiety and depression symptoms of university students, especially for male students and students who are not directly exposed to the highest levels of the epidemic. During the pandemic, university students showed a high level of interest in receiving psychological knowledge and interventions, especially for information that could help them alleviate negative psychological effects (Wang Z. et al., 2020). As adaptability has been identified as a key factor in protecting students from anxiety and depression during the Covid-19 pandemic (Zhang et al., 2021), fostering psychological flexibility could be a beneficial approach to addressing the negative consequences of pandemic on mental health (Kashdan and Rottenberg, 2010). Psychological flexibility is defined as the capacity to adapt one's behavior in a manner that incorporates conscious and open contact with thoughts and feelings (Scott et al., 2014). In the context of the pandemic, recent research has demonstrated that psychological flexibility plays a moderating role in the effects of the lockdown, relating to better mental health in a wide range of contexts, and that inflexibility is a risk factor for anxiety and depression (Hayes et al., 2006; Pakenham et al., 2020). This indicates that government, university, and mental health counselors should pay more attention to students with symptoms of anxiety and depression, as well as their related cognitive issues. It also essential for authorities to provide psychological knowledge, such as common symptoms of anxiety and depression, methods for alleviating negative psychological effects, and contact information for counseling services, to university students. Our findings highlight the increased burden of remote learning for the mental health of certain individuals (e.g., male students, students with pre-existing symptoms of anxiety and depression, and residents of areas with lower epidemic levels and healthcare budgets), which should be taken into account as well.

There are some limitations of the current study. First, data collection was completed by online research with self-report scales, which may limit the objectivity of the data. Second, although the university students came from more than 34 provinces, they all came from China, so the generalizability to populations in other cultures should be made with caution. Future research should replicate this model in other regions of the world. Third, in order to avoid carry-over effects and potential boredom from survey repetition, our participants completed two different measures of anxiety and depression at the two time points. Although both sets of measurements are highly reliable and validated by previous research (e.g., Liu et al., 2021), it can be problematic when studies use different measurements to measure the same construct (e.g., Feuer et al., 1999). However, based on recommendations to calculate outcome variables based on a common metric (Marcoulides and Grimm, 2017), we applied the standardized values of these two measurements (e.g., Ayubi et al., 2021). We acknowledge, however, that this was a limiting factor for our conclusions.

## Conclusion

The present longitudinal study investigated the changes in the mental health status of college students in mainland China during the epidemic of COVID-19. The findings confirmed a significant increase in anxiety and depression among students. Results suggested that the increase of anxiety and depression was related to gender, pre-existing levels of anxiety and depression, and the severity of the epidemic in their geographic region.

## Data Availability Statement

The datasets presented in this study can be found in online repositories. The names of the repository/repositories and accession number(s) can be found below: https://osf.io/64aw7/?view_only=fc4a64aca2a7481a866434d7da631a9d.

## Ethics Statement

The studies involving human participants were reviewed and approved by Qingdao University. Written informed consent to participate in this study was provided by the participants' legal guardian/next of kin.

## Author Contributions

SW and KZ: conceived and designed the survey, performed the survey, and contributed materials/analysis tools. KZ and ZH: analyzed the data. SW, KZ, EP-S, ZH, YJ, and XC: wrote the paper. KZ, EP-S, ZH, YJ, and XC: literature research. All authors contributed to the article and approved the submitted version.

## Conflict of Interest

The authors declare that the research was conducted in the absence of any commercial or financial relationships that could be construed as a potential conflict of interest.
